# Confidence intervals for putative quantitative trait loci – development and applications of new linkage methods

**DOI:** 10.1186/1753-6561-1-s1-s91

**Published:** 2007-12-18

**Authors:** Charalampos Papachristou, Mark Abney, Shili Lin

**Affiliations:** 1 Department of Human Genetics, University of Chicago, 920 East 57^th ^Street, Chicago, Illinois 60637, USA; 2 Department of Statistics, The Ohio State University, 1958 Neil Avenue, Columbus, Ohio 43210, USA

## Abstract

A new method for constructing confidence intervals for the location of putative genes regulating expression levels (quantitative traits) is proposed. This method is suitable for the "intermediate" fine-mapping step usually performed between the initial whole-genome screening and the follow-up fine mapping step as a means of reducing the size of the region where the latter is performed. Assuming the existence of a single quantitative trait locus (QTL) in the region/chromosome identified by the genome scan, the method constructs a confidence region for its true position by testing each location in the chromosome to see if it can be the trait locus. We applied our method to the gene expression data from Problem 1 of Genetic Analysis Workshop 15 (GAW15) data, focusing on 25 genes that have previously been shown to share common regulating factor(s) on chromosome 14. Our results pointed to the same region on chromosome 14 for 13 of the gene expressions studied, not only partially reproducing the results of the previous analysis, but also yielding 95% confidence regions for the regulatory quantitative trait loci. Moreover, we identified three regions, one on each of the chromosomes 3, 9, and 13, which potentially harbor additional common QTLs for several of the original gene expressions.

## Background

The identification of variants (single-nucleotide polymorphisms, or SNPs)/genes that contribute to variation of a quantitative trait is of great importance for understanding these traits. Although there is a wealth of methods available for detecting such quantitative trait loci (QTLs), most of them are designed to provide evidence in favor of or against the existence of a QTL. As such, these methods can only point to the broad genomic regions where the putative loci lie, but they do not readily provide confidence regions for the actual positions of the trait genes. Such confidence intervals are useful for designing follow-up studies, after preliminary linkage signals have been detected, because they can significantly reduce the number of candidate genes, thereby making follow-up studies more time- and cost-efficient. Finally, an additional challenge that almost all current approaches are faced with is the need for multiplicity adjustment for the number of hypothesis tests performed. This is a rather formidable task to undertake, given the complex dependencies that data from genetic studies experience.

We propose a new method that can be used in the "intermediate" fine-mapping step [1,2] between whole-genome screening and follow-up studies to provide confidence regions for the positions of putative trait regulating loci identified by the former. Under the assumption of a single QTL on the chromosome of interest, we test every location in the region to see if it can possibly be the position of the putative locus. We then derive a confidence region for the true position of the gene by aggregating all locations for which this hypothesis is not rejected. By controlling the level at which the hypothesis tests are performed, one can easily obtain confidence regions with a desired coverage probability.

We applied our method to the gene expression (GE) data of 194 individuals from the Centre d'Etude du Polymorphism Humain (CEPH) Utah families of European descent (GAW15 Problem 1). From a total of more than 3500 GEs available, we decided to focus on 25 that have been suggested to be (co-)regulated by variants located on chromosome 14 [3]. More specifically, we treated each GE as a quantitative trait and used our approach to construct confidence regions for the loci contributing to its variation.

## Methods

### The hypotheses and the test statistic

For an arbitrary location *τ *in the genome consider testing the following hypotheses:

*H*_0 _: *τ *= *τ *vs. *H*_0 _: *τ *≠ *τ*,

where τ* is the true location of the QTL controlling the expression level of a continuous phenotype. If we perform the above hypothesis test at an *α*-level, then it is easily seen that the collection of all loci *τ *that the null hypothesis is not rejected forms an 1 - *α *confidence region for the location of the QTL. In addition to allowing us to derive confidence regions with known statistical properties, this formulation of the null and alternative hypothesis helps circumvent the need for multiplicity adjustment for the number of locations tested.

In order to test the above hypotheses, we made use of the method of the squared differences (SQD) of phenotypic values of sib pairs introduced by Haseman and Elston (HE) [4]. In brief, let us assume that we have phenotypic values of a quantitative trait for *n *sib-pairs, and let *y*_*i *_be the squared difference of the expressions of the two siblings in the *i*^th ^family. In addition, define *π*_*i *_to be the proportion of alleles shared identical by descent (IBD) by the sib pair at the trait locus. HE [4] proved that under the regression model

*Ey*_*i *_= *β*_0 _+ *β*_1_*π*_*i*_,

and assuming null dominance effect, or fairly large sample size, the coefficient *β*_1 _is simply -2$\sigma_{a}^{2}$, where $\sigma_{a}^{2}$ is the additive variance component attributed to the trait locus. Fulker and Cardon [5] demonstrated that the same holds if *π*_*i *_is substituted by *π*_*i*_(*G*_*i*_), the expected number of alleles shared IBD *given *the marker genotypes (*G*_*i*_) of the family. Based on this observation, one can easily test Eq. (1) by simply testing the following hypotheses for each locus *τ*

$$H_{0}:\beta_{1\tau }=-2\sigma_{a}^{2}\;vs.\;\beta_{1\tau }\rangle -2\sigma_{a}^{2},$$

where *β*_1*τ *_is the regression coefficient of the SQDs *y *values, on the *π*_*τ*_*(G) *values, the observed IBD sharing by the sib pair at the locus *τ*. Thus, in order to construct the confidence region, we only need to estimate the additive effect of the trait locus. Such an estimate can be obtained from the data themselves after a linkage signal has been established, i.e., the maximum test statistic used in the preliminary analysis exceeds a certain pre-chosen threshold. Assuming that the total number of sib pairs (*n*) is sufficiently large, we can randomly split the data into two groups of *n*_1 _and *n*_2 _= *n *- *n*_1 _pairs. Using the data from the first group, we can estimate the additive genetic variance as ${\hat{\sigma }}_{a}^{2}=\frac{1}{2}\beta_{1r_{\max}}$, where ${\hat{\beta }}_{1r_{\max}}$ is the estimate of the regression coefficient of the SQD values on the observed IBD sharing at the locus *τ*_*max*_, the location where the maximum test statistic of the whole genome screening occurred. Then, based on the data from the second group and for each location *τ *on the genome, we fit the regression in Eq. (2) to obtain the coefficients ${\hat{\beta }}_{1r}$. Finally, we test

$$H_{0}:\beta_{1\tau }=\beta_{1\tau_{\max}}\;vs.\;\beta_{1\tau }\rangle \beta_{1\tau_{\max}}$$

to determine whether *τ *should be included in the confidence region for the location of the putative trait contributing gene.

### Data and phenotypes

Our data consisted of GE measurements on 3554 genes for 14 three-generational CEPH families, averaging 14 members per pedigree for a total of 194 people. We chose to analyze the GEs of 25 genes that have been suggested to be (co-)regulated by one or possibly multiple SNPs located on a small genomic region of 5 cM toward the end of chromosome 14 [3]. Genotypes on about 2800 SNPs densely covering the whole genome (on average 1 SNP/cM) were also available for all individuals. Because most of the families were multigenerational with more than two children, we extracted all possible combinations of sib pairs, resulting in a sample of 376 pairs. Note that this procedure does not yield an independent sample. We could select one sib pair from each family and exclude it from the analysis to remove this dependency [6]. However, previous experience with the CSI version for binary traits [7] suggests that this dependency has a minimal effect on the confidence regions. Because we expected the CSI-QTL method to have a similar behavior as its counterpart for binary traits, we opted to include all the pairs in the analysis.

### Analyses

Each GE of interest was analyzed according to the following two-stage protocol. First, using the HE test statistic we scanned the whole autosomal genome, 22 chromosomes, to uncover chromosomes that potentially harbor regulating factors. In an attempt to hold the false-discovery rate at a low level, we selected for further analysis only those chromosomes that had a minimum observed standardized score for *β*_1 _of less than -3.09 (pointwise significant level of 0.001). In the second stage, we focused only on the chromosome(s) that were identified on the preliminary analysis and applied our proposed method to construct 95% confidence regions (CRs) for the true location of the identified putative QTL. Although the sample size of 376 sib pairs seems to be sufficiently large for each stage of the analysis, splitting them into two groups as we described earlier would lead to too small a sample size for either. Thus, we decided to use all the sib pairs for both estimating the additive variance and constructing the CRs. We expect this to result in relatively conservative intervals with true coverage probability higher than their nominal value.

## Results

**Figure 1:**
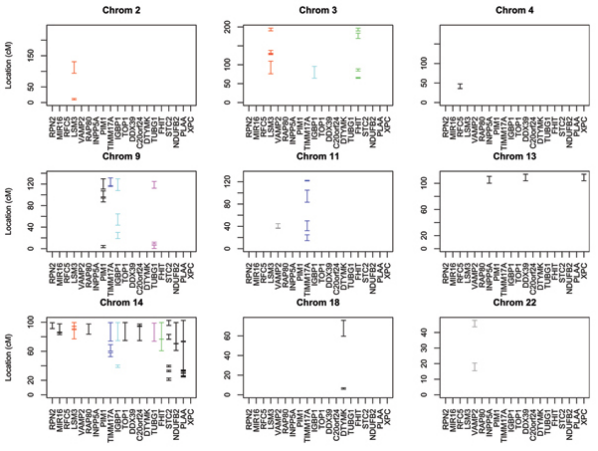
**95% confidence regions for the 20 GEs that pass the initial genome screening**. For each plot, the horizontal axis gives the name of each gene. The vertical axis gives the length of the corresponding chromosomes, while the line segments in the graph display the CRs on each chromosome. GEs with CRs on only one chromosome are plotted with black color, while those with CRs spanning multiple chromosomes are color-coded for easier identification.

Of the 25 GEs studied, only 20 of them revealed signals in the preliminary scan on at least one chromosome. For the majority (14) of the GEs, the genome screening suggested the existence of a single chromosome housing a putative QTL, while for half (3) of the remaining ones, it identified two chromosomes. For the last three of them it pointed to joint regulatory loci in three different chromosomes. In Figure 1 we plotted all the resulting non-null 95% CRs. The 14 GEs with CRs on a single chromosome are plotted in black color, while the remaining GEs with CRs spanning multiple chromosomes are color-coded so as to be easily identified across chromosomes. As it can be seen from the figure, the resulting CRs were not uniformly distributed across the genome. They were located on a total of nine chromosomes, with most of them clustered at chromosome 14, possibly implying common co-regulating loci located on a ~24 cM region at the end of the chromosome. All CRs on chromosome 14 included the 5-Mb region 14q32, thereby reconfirming the findings of Morley et al. [3] that found evidence of *trans*-acting co-regulators in that region. Furthermore, the CRs on three additional chromosomes (3, 9, and 13) experience a significant overlap, suggesting the potential existence of additional co-regulating factors for several of the GEs investigated.

Our results do not provide strong evidence to support the existence of *cis*-regulating factors because for almost all of the GEs, the constructed CRs are located on different chromosomes than the one on which the gene under investigation resides. The only exception is gene *FHIT*, which is located on chromosome 3 about 70 cM from the beginning of the chromosome. As we can see from the figure (green line) its CR includes the segment (64 cM, 66.5 cM) on the same chromosome, suggesting a possible *cis*-regulating locus. Finally, although the CR for gene *LSM3 *(chromosome 3, at 3.1 cM) included genomic segments on chromosome 3, these segments were at least 70 cM away from its location, and hence do not provide strong support for a *cis*-acting regulator.

## Discussion

We have described a new method that can be used as a tool in the "intermediate" fine-mapping step of linkage analysis studies for providing confidence regions for the location of putative QTLs after linkage signal has been detected. Application of our method to the gene expression data from the CEPH families demonstrated that it can successfully narrow the locations of regulatory regions of expression levels of genes. By focusing on 25 GEs, we were able to corroborate the previous finding of a 5-cM regulatory region on chromosome 14 for multiple gene expression traits. Furthermore, we found some evidence for the existence of three additional regions that may also contain loci that co-regulate the expression levels of several of the genes studied.

Although we are highly encouraged by the concordance between our results and those of previous analyses on the same data, the resulting intervals were slightly wider than what one might desire, averaging about 25 cM. A contributing factor might be that we estimated the additive variance component of the identified QTL from the data themselves. As such, this estimate was correlated with the data, leading to slightly wider intervals. Using two independent samples, one for the estimation of the additive variance and one for the construction of the CRs, will help moderate this effect and likely lead to greater precision. However, in a real situation it may not be possible to obtain two independent samples. In such a case, one may employ alternative approaches to estimate the value of the additive variance, such as variance-components estimation methods [8]. We intend to investigate such alternatives in the future in hopes of achieving greater localization of the positions of the putative genes.

The current formulation of the method assumes negligible dominance variance at the trait locus identified by the preliminary scan. Although in many instances this additive model may be a good approximation [9], there might be situations in which this model may not be valid. In such a case, ignoring existing dominance variance would result in an inflated estimate of the additive variance component. This inflation in turn may result in narrower intervals with true coverage probability lower than the nominal coverage that the intervals are reported to have. Nevertheless, this would only be an issue for relatively small sample sizes, because the bias of the additive variance estimate tends approach to zero as the number of sib pairs increases [4]. Given the fairly large sample size we had in our application, we expect that any effect from violation of this assumption to be minimal.

An issue with this two-step procedure is the fact the additive genetic variance is estimated only after a linkage signal has been established. Thus, it is very likely to be biased upwards, especially when the sample size is relatively small [2,10,11]. This bias may result in intervals with lower coverage probability than their nominal coverage probability. Using a bootstrap approach to obtain less biased estimates [11] may help moderate the effect of this bias. However, Papachristou and Lin [7] showed that in the case of binary traits, even though the genetic parameters may be biased, the true coverage of the resulting CSI intervals stays above their nominal level. This behavior is partially due to the fact that the CRs are constructed after the preliminary analysis gives strong signal in the region, thereby increasing the chances of the interval to capture the identified putative locus [7]. We do anticipate the CSI-QTL procedure to experience similar behavior as in the case of binary traits, but more investigation is needed to substantiate this claim.

Finally, throughout this current paper we assumed the existence of at most one trait contributing locus on the region/chromosome identified from the preliminary analysis, and used our proposed method to construct a CR for its true location. If this assumption is violated, the coverage probability of the resulting interval is not guaranteed to be close to the nominal value for any of the putative trait loci in the region. In fact, the actual coverage of the derived CR would depend on both the distance between the trait loci and their relative contribution to the quantitative phenotype [12]. An extension of our proposed method that accommodates joint localization of multiple loci would be of great interest and we intend to explore such approaches in the future.

## Competing interests

The author(s) declare that they have no competing interests.
